# Factors influencing speech language pathologists’ and audiologists’ decision to pursue postgraduate studies in South Africa

**DOI:** 10.4102/sajcd.v68i1.796

**Published:** 2021-05-18

**Authors:** Ben Sebothoma, Khetsiwe Masuku, Nomfundo Moroe

**Affiliations:** 1 Department of Speech Pathology and Audiology, Faculty of Humanities, University of the Witwatersrand, Johannesburg, South Africa

**Keywords:** speech language pathologist, audiologist, influencing factors, postgraduate studies, postgraduate degree, South Africa

## Abstract

**Background:**

Factors that influence various professionals to pursue postgraduate studies have been investigated. However, there is a dearth of evidence of factors that influence South African speech language pathologists (SLPs) and audiologists (As) to pursue their postgraduate studies.

**Objective:**

Therefore, this study aimed to determine factors that influence the decision of South African SLPs and As to pursue postgraduate studies and potential barriers to this pursuit.

**Method:**

A quantitative survey research methodology with a cross-sectional research design was adopted, where a 21-item web-based survey was used to survey 127 speech therapists, As and speech therapists and As from across the country. All participants were qualified and registered with the Health Professionals Council of South Africa (HPCSA). Ethical clearance and permission from relevant stakeholders were obtained. Data were analysed descriptively.

**Results:**

The findings suggested that over half of the participants pursued their postgraduate studies to fulfil a personal goal and improve their knowledge, whilst others did it to develop expertise and for job opportunities. Participants highlighted that a lack of time and funding, heavy workloads and bad experiences during their undergraduate studies were the main barriers to pursuing postgraduate studies. Whilst community service was not a barrier per se, participants felt that it delayed them from beginning their postgraduate studies immediately.

**Conclusion:**

The findings of this study highlighted the urgent need for institutions of higher learning, government and other stakeholders to provide the necessary support for SLPs and As in South Africa to pursue postgraduate studies.

## Introduction

Speech language pathologists (SLPs) and audiologists (As) are health professionals that deal with the assessment and treatment of patients with communication difficulties (including hearing loss) and swallowing disorders (Gillam & Gillam, 2011). Literature suggests that people who pursue a career in speech language pathology or audiology (SLP or A) are often motivated by the desire to help people. For example, Du Plessis (2018) and Stone and Pellowski (2016) found that 74% and 70%, respectively, of SLP and A students chose their profession because of this desire. Whilst the reasons for the pursuit of SLP and A as a profession are clear in the literature, the reasons for the pursuit of postgraduate studies in SLP and A, especially in a South African context, are not clear. Therefore, this study aimed to explore factors that influence students’ and clinicians’ decision to pursue postgraduate studies in SLP and A.

Postgraduate education is increasingly becoming important in many countries worldwide. As a result, institutions of higher learning are encouraged to develop strategies to increase the number of their postgraduate students (McCallin & Nayar, 2012; National Development Plan [NDP], 2012). In low- and middle-income countries, such as South Africa, postgraduate education is recognised as one way of enhancing economic growth (NDP, 2012) and challenging the colonial epistemology through the production of contextually relevant knowledge (Khoza-Shangase & Mophosho, 2018; Mdlalo, Flack, & Joubert, 2019; Pascoe & Norman, 2011; Pillay & Kathard, 2015). This is particularly important for the SLP and A profession, which has always been governed by first world knowledge (Khoza-Shangase & Mophosho, 2018). Consequently, South Africa, through the NDP, intends to produce as many as 100 doctoral graduates per one million per year by the end of 2030 (NDP, 2012).

Whilst obtaining a postgraduate degree is important because of its prospective transformational and economic benefits, postgraduate students continue to face a myriad of challenges that may threaten the achievement of initiatives, which address socioeconomic factors (Albertyn, Kapp, & Bitzer, 2008). For example, postgraduate students often experience financial difficulties or a lack of family time, whilst others struggle with writing skills (Abdullah & Evans, 2012; Govender et al., 2018; Yasmin et al., 2018). Despite these known challenges, literature suggests that there are students who persistently pursue their postgraduate studies (Cobbing et al., 2017). The aim of the study was therefore to investigate the potential facilitators and barriers to the pursuit of postgraduate studies. It is hoped that the results of this study may be of benefit to policymakers in South African Universities and health service providers and to SLP, audiologist and speech pathologists, audiologist and SLPs and audiologist.

The motivation to pursue postgraduate studies may not necessarily be in line with national programmes and vision. Literature suggests that people pursue postgraduate studies for different reasons. For example, Jasińska-Stroschein, Kurczewska and Orszulak-Michalak (2017) in a Polish study conducted with 414 pharmacy students found that pharmacy students pursued their postgraduate studies to acquire a promotion at work. A total of 425 physiotherapy students in South Africa and 1632 nursing students in Australia indicated that they pursued postgraduate studies because they wanted to develop their clinical expertise, fulfil personal goals and improve patient care (Cobbing et al., 2017; Ng, Eley & Tuckett, 2016). A study conducted with non-medical science students in Korea by Jung and Lee (2019) indicated that students enrolled into postgraduate studies if they were satisfied with their undergraduate education.

In the profession of SLP and A, motivations to pursue postgraduate studies were found to be different from other professions. Internationally, a postgraduate degree such as a clinical doctorate in audiology (e.g. AuD) or master’s degree in SLP is a required minimum qualification to become a licensed clinician (Martin & Clark, 2015). The American Speech Hearing Association (ASHA) specifically stipulates that SLPs require a minimum of a master’s degree, whilst audiologists require a clinical doctorate to be certified as clinically competent (CCC-SLP/A) (ASHA, 2007). In Australia, a postgraduate degree (i.e. Master of Clinical Audiology) is regarded as an entry level to practice as an audiologist (Audiology Australia, 2013).

Therefore, the motivation and decision to pursue postgraduate studies in SLP and A in countries such as the United States of America and Australia are predicated on the career trajectory to become a licensed clinician. However, in a South African context, where an undergraduate 4-year degree (e.g. BA, BSc) in SLP and A is a sufficient qualification to practice as a clinician in any clinical setting (Health Professions Council of South Africa [HPCSA], 2011; South African Speech Language Hearing Association [SASLHA] Ethics and Standards, 2010), factors that influence a graduate’s decision to pursue postgraduate studies are not well studied. Hence, there is a need for this study.

## Methods

### Aim

The aim of this study was to determine the factors that influence the decision of South African SLPs and As to pursue postgraduate studies and potential barriers to this pursuit.

### Research design

In this study, we employed a quantitative survey research methodology with a cross-sectional research design (Creswell, 2013). A quantitative approach was adopted because the study sought to quantify the number of students who return for postgraduate studies. In this study, a web-based survey research was utilised as it allowed the researchers to gather data from speech and hearing professionals across South Africa (Khoza-Shangase & Masondo, 2020). This method has previously been used successfully in a South African context with both SLPs and As (Khoza-Shangase & Masondo, 2020; Khoza-Shangase, Sebothoma, & Seedat, 2019; Makhoba & Joseph, 2016; Seedat, Khoza-Shangase, & Sebothoma, 2018), because it enabled the researchers to gain access to individuals in distant locations (Wright, 2005).

### Participants

A non-probability purposive sampling strategy was used to recruit potential participants where specific inclusion and exclusion were adopted (Leedy & Ormrod, 2013). Participants in this study had to be registered with the HPCSA and SALSHA and/or South African Association of Audiology (SAAA). The SASLHA and the SAAA were contacted to gain access to their members’ contact details in order to distribute the survey link to their members. A total of 127 participants (28 As, 43 SLPs and 56 SLPs and As) who were registered with the HPCSA and working in different settings completed the survey. Descriptive statistics were used to analyse the data (Ali & Bhaskar, 2016). Percentages and graphs were used to summarise and describe all the sections of the questionnaire.

### Data collection and instrument

The research questionnaire used in this study was developed according to the aims and objectives of the study. The questionnaire was developed based on previous research (Cobbing et al., 2017; Hammond et al., 2017; Jasińska-Stroschein et al., 2017; Jung & Lee, 2019), and items were generated to suit the context of this study. The items on the questionnaire were predominantly closed-ended questions, with multiple choice answers. The questionnaire comprised of 21 questions and three sections, namely the demographic characteristics of the participants, postgraduate status and factors influencing the decision to pursue postgraduate studies. The survey questionnaire was piloted with two professionals, one SLP and one A using the same methodology used in the main study. The purpose of the pilot study was to determine the content and face validity of the questionnaire (Leedy & Ormrod, 2015). The two professionals (SLP and A) were asked to comment on the content and to determine whether the content of the questionnaire is in line with the objective of the study. In addition, the two professionals were asked to comment whether at face value, the questionnaire has captured what is being investigated. The pilot study showed that the questionnaire was appropriate and could be completed in approximately 10 min. The final questionnaire was uploaded on Survey Monkey^TM^, which was used to create the survey. The participants received an email from their associations (SASLHA and SAAA) containing the link of questionnaire. A 3-month cut-off time was set for participants to respond to the survey.

### Procedure

Permission to conduct the study was obtained from the professional associations (SAAA and SASLHA). Once permissions and ethical clearance had been obtained from the relevant authorities, a survey questionnaire was distributed to South African SLPs who are registered members of SAAA and SASLHA, using SurveyMonkey^®^. The survey questionnaire was accompanied by an information sheet, which detailed the purpose and nature of the study. All participants provided informed consent by completing the survey.

## Results

### Description of participants

**FIGURE 1:**
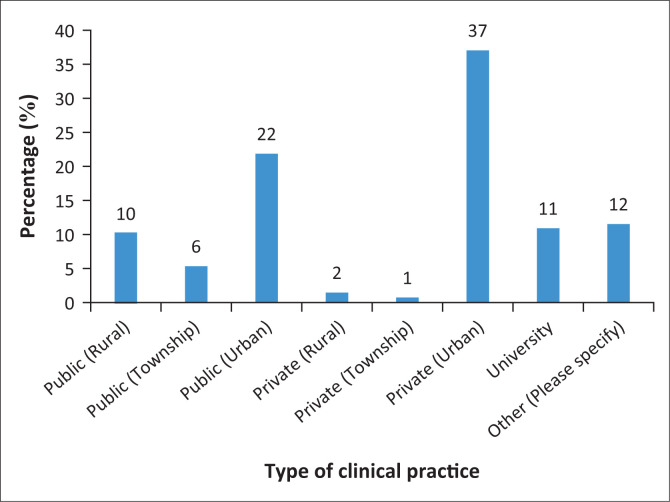
Type of practice setting.

**FIGURE 2:**
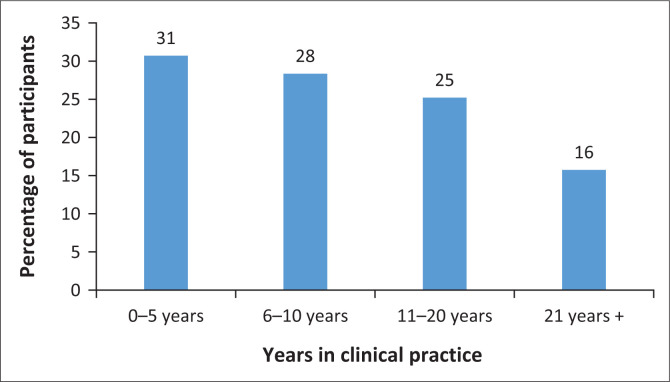
Number of years spent in clinical practice.

A total of 127 participants responded to the survey, with the majority of the participants (*n* = 75; 59%) working in urban areas (Figure 1). Majority of the participants (57%) were white practitioners, whilst 23% of the participants were black African practitioners. Participants varied in terms of how many years they had been in clinical practice (Figure 2). Almost half of the participants (44%) were dually qualified (SLP and A), whilst the rest were either SLPs or As. The majority of the participants (*n* = 72; 57%) indicated an undergraduate degree (e.g. BA or BSc) in SLP and/or audiology as the highest level of qualification obtained with the rest possessing a postgraduate degree.

### The pursuit of postgraduate studies

**FIGURE 3:**
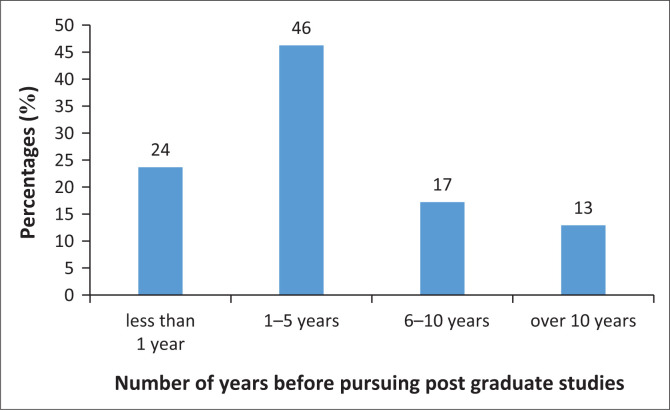
Number of years before the pursuit of postgraduate studies.

Only a minority of participants (37%) were currently pursuing postgraduate studies. Of those, 36% were pursuing a master’s in research, 23% a research doctorate (e.g. PhD), 9% coursework masters, 1% clinical doctorate (AuD) and 31% indicated ‘other’. A significant number of participants (30%) were pursuing postgraduate studies in other professions such as psychology (e.g. honours) or business (e.g. business administration). Of the participants who had completed or were currently pursuing their postgraduate studies, almost half of them (46%) had waited between 1 and 5 years before registering for their postgraduate studies (Figure 3). There are various reasons why participants waited for that specific period, including personal, a lack of funding, being impeded by community service year, feeling uncertain about the research topic, not seeing the value of postgraduate studies or wanting to take a break. Even though the majority of participants stated their reasons for waiting for a certain period before pursuing their postgraduate studies, 44% of the participants responded ‘other’ to why they waited for a specific period.

### Factors influencing students’ decision to pursue postgraduate studies

**FIGURE 4:**
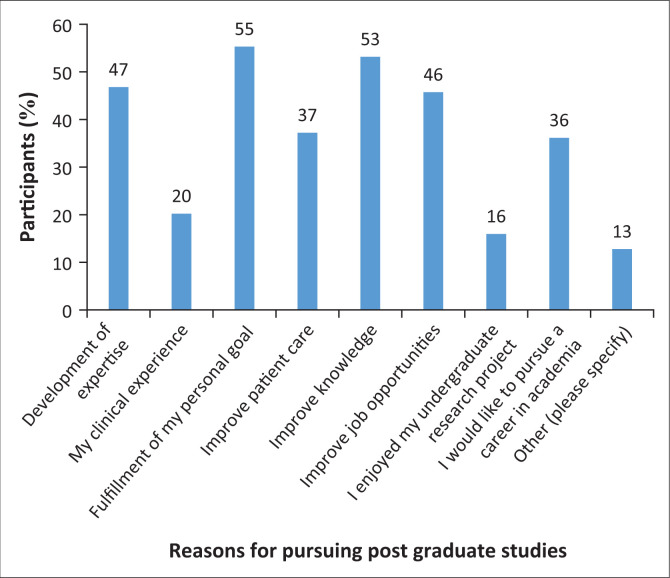
Factors influencing speech language pathologists’ and audiologists’ decision to pursue postgraduate studies.

Participants were asked to indicate the factors that influenced their decision to pursue postgraduate studies. The primary factors were fulfilment of a personal goal (55%) and improving their knowledge (53%). Almost half of the participants indicated that the need for the development of expertise (47%) and improving job opportunities (46%) also influenced their decision to take up postgraduate studies. Very few participants indicated that they were influenced by other factors, as depicted (Figure 4). The majority of the participants (69%) indicated that their community service experience did not influence their decision to pursue postgraduate studies; instead, some highlighted that it had delayed them from enrolling immediately after completing their undergraduate studies.

### Barriers to pursuing postgraduate studies

Participants were asked to outline barriers that impeded them from pursuing postgraduate studies. The primary barriers indicated were a lack of time because of family commitments, heavy workloads and a lack of funding:

‘Single mom needed to work to support my children and had neither the finances nor the time to further my studies.’ (Participant 1, SLTA, Female)

‘The biggest issue is the time commitment. I’m interested in research, but I am also very busy and involved in other professional and other activities. I am often too tired in the evenings to do anything academic. I also have financial and other insecurity; my husband is unemployed and my salary must cover the expenses for him, my son and his girlfriend, who are not yet financially independent. If I could do it at my own pace I would be more inclined to register for post-graduate studies, because the risk of having to deregister because of time constraints would no longer be there.’ (Participant 2, SLTA, Female)

‘Time and finances – I worry about having enough time to maintain my current workload and income yet still be able to give enough time to completing a postgraduate degree and making it worthwhile.’ (Participant 3, SLTA, Female)

Furthermore, participants indicated that they were not pursuing postgraduate studies because there were no financial and/or clinical benefits of doing so in South Africa. These participants believed that attending seminars or continuing professional development (CPD) activities was equally beneficial. Some participants highlighted that their ‘bad experiences’ during their undergraduate years at university made them unwilling to return and pursue their postgraduate studies:

‘I believe in obtaining a variety of skills that are often not included in postgraduate programmes. I would rather attend workshops and seminars that interest me.’ (Participant 4, SLTA, Female)

‘I have been working for 26 years and have specialised in the paediatric scholastic population. I feel that with regular CPD activities I am able to maintain my knowledge without having to do a postgraduate degree. My undergraduate studies were so stressful that I vowed never to study again, and to date I have not had the desire to go back to university. My life is also very busy with three children and I do not think I have sufficient time at the moment.’ (Participant 5, SLTA, Female)

Some of the participants reported that they were not pursuing postgraduate studies, particularly in their community service year because they prohibited by ‘a policy’:

‘[*W*]anted to wait 1 year, to rest after 4 years of studies, and focus on my community service year.’(Participant 6, SLTA, Female).

‘I had to complete my community service first because I was told I couldn’t do my masters in my community service because it is against the HPCSA the policy.’ (Participant 7, SLTA, Female)

Lastly, low marks obtained during undergraduate (i.e. research report marks) also made SLPs and As reluctant to pursue postgraduate studies.

## Discussion

The purpose of this study was to determine factors that influence South African SLPs’ and As’ decision to pursue postgraduate studies, and potential barriers to this pursuit. Findings from this study suggest that there are various factors that influence South African SLPs’ and As’ decision to pursue postgraduate studies. South African SLPs and As and SLTAs indicated that they pursued their postgraduate studies to fulfil their personal goals and to improve their knowledge. The findings of this study corroborate the findings of Hoffman and Julie (2012) who found that 57.8% of the students in the faculty of community health science who worked in the areas of nursing, public health, social work, physiotherapy, human ecology, psychology, occupational therapy, sports and recreational science and human ecology and natural medicine pursued their postgraduate studies because of the need to improve their knowledge in the field.

Whilst South African SLPs and As primarily pursued their postgraduate studies to improve their knowledge and opportunities, approximately half (47%) of the SLPs and As indicated that they pursue postgraduate studies to develop their expertise in a specific area and improve their job opportunities. These findings were consistent with findings from a study by Cobbing et al. (2017), who also found that some South African physiotherapists pursued postgraduate studies to develop their expertise in a specific area. These findings suggest that SLP and As see value and relevance of postgraduate studies in clinical practice. Therefore, these findings raise important curriculum implication. Institutions of higher learning may need to also establish postgraduate programmes that placed greater emphasis on developing or improving specific expertise. Cobbings et al. (2017) also reported that physiotherapists were likely to pursue postgraduate studies that placed an emphasis on acquisition of clinical skills.

It was assuring to find that participants constantly received information on postgraduate studies. Participants indicated that information on postgraduate opportunities was being disseminated by professional associations such as SAAA and SASHLA, and some participants had personally been contacted by academics to pursue their postgraduate studies. Current authors encourage further and wider distribution of information on postgraduate studies, which include transparent information on available funding opportunities. Given that not all SPL and As are affiliated with SAAA and/or SASLHA, there must be an effort to reach those who are not affiliated with these associations and those who do not have access to internet. For example, professional associations such as SAAA and/or SASLHA can collaborate with institutions of higher learning through their alumni programmes to reach wider audience.

The biggest barriers to pursuing postgraduate studies reported in this study were lack of time, funding opportunity and family commitment. Cobbing et al. (2017) and Havenga and Sengane (2018), also found that participants described lack of time, funding and family commitments as the main barriers to pursuing postgraduate studies. Mutula (2011) suggested that funding opportunities for postgraduate students remain a problem across the African continent. Cleary et al. (2011) believed that the high attrition rates of postgraduate students are because of financial difficulties. These findings raise implications for resource distribution and allocations. Institutions of higher learning should create awareness on available funding opportunities for postgraduate studies. Given that postgraduate studies provide an opportunity to improve knowledge and skills, thus contributing towards the quality service offered to patients, it would benefit the health facilities such as hospitals to create time and support for clinicians who want to pursue postgraduate studies, if financial support is available.

Some participants in this study highlighted ‘bad experiences’ during their undergraduate years as one of the reasons they are not pursuing postgraduate studies. In a study conducted by Jung and Lee (2019), satisfaction with undergraduate study had a positive effect on enrolment in postgraduate studies. In this study, participants did not specify what constituted and contributed to their ‘bad experience’. It is not known whether the individual factors described in studies conducted by Seabi, Seedat, Khoza-Shangase and Sullivan (2012) and Tanga and Maphosa (2018) or combination of these factors contributed to the ‘bad experiences’. Therefore, there is a need for further studies to explore specific factors that contribute to ‘bad experiences’ of students during their undergraduate year. These findings raise implications for support of students during their undergraduate studies. Undergraduate programmes must create a conducive environment, provide adequate support and positive experience for undergraduate students so that they are most likely to consider returning for postgraduate studies.

An interesting finding in this study was that some SLP and As participants stated that an HPCSA policy impeded them from studying during their community service year. Current authors searched for the policy on the HPCSA website but could not find such document. Despite the lack of available policy, most participants (69%) indicated that their community service experience did not influence their decision to pursue postgraduate studies. In fact, some participants highlighted that their community service year has delayed their commencement of their postgraduate studies. Burman et al. (2019) argued that community service in South Africa, which focusses primarily on clinical exposure for new graduates, makes it difficult for professionals to maintain a research interest. Firstly, the authors of this study propose that a policy on community services and studies be transparent and easily accessible. If such policy exists and it impedes graduates from pursuing postgraduate, based on current findings, this policy requires a review. Hanson, Paulsen and Pascarella (2016) argued that in order to increase the number of postgraduate students, policy makers must understand what motivates students to pursue their postgraduate studies. Therefore, as Burman et al. (2019) showed, these findings suggest the need for restructuring community service in order to increase research interest in graduates.

Finally, obtaining low research report scores during an undergraduate year was also reported as a potential barrier to pursuing postgraduate studies. Given that there is no strong association between undergraduate performance and postgraduate success (Woloschuk, McLauglin, & Wright, 2010), these findings suggest that institutions of higher learning need to reconsider their entry requirement for postgraduate studies. Secondly, institutions of higher learning should consider pre-Masters and pre-Doctoral programs, particularly to support and accommodate potential postgraduate students who may not necessarily meet their entry requirement.

### Study limitations and recommendations

The recruitment method used in this study permitted access only to participants registered with SAAA and SASLHA. Therefore, SLP and As who were not affiliated with these associations could not participate in the study. Future studies need to use recruitment methods that allow for wider reach of participants, including those who are not affiliated with these associations. In addition, participants without access to internet could not participate in this study. As a result of the limitations of the recruitment method used, it was not possible to accurately calculate the response rate. As some participants were affiliated with both associations, it was not possible to tease out which of the participants were affiliated with one or both associations. Previous studies that used similar method could not calculate the response rate (Khoza-Shangase et al., 2020; Makhoba & Joseph, 2016; Seedat et al., 2018). Future studies should include a question that requires participants to indicate their affiliations. Future studies should also consider employing a qualitative research, which will explore the results further and provide an in-depth information (Cobbing et al., 2017).

## Conclusion

These findings highlighted the need for support of SLPs and As who intend to pursue their postgraduate studies. Various stakeholders need to provide the necessary support to enable clinicians to pursue their postgraduate studies. This support is crucial and urgent given that postgraduate study in countries such as South Africa is linked with the transformation and decolonisation of the two professions. For example, in South Africa, there is an extreme shortage of black academics and the content of the curriculum is still based on euro-western epistemology (Khoza-Shangase & Mophosho, 2018). Increasing the number of postgraduate students in SLP and A may result in an increased number of black people in academia. However, institutions of higher learning need to create a supportive environment that will motivate students to pursue their postgraduate studies. These findings also highlighted the need for policy review on community service for both SLP and A.
